# The effects of a pro-active integrated care intervention for frail community-dwelling older people: a quasi-experimental study with the GP-practice as single entry point

**DOI:** 10.1186/s12877-016-0214-5

**Published:** 2016-02-15

**Authors:** Wilhelmina Mijntje Looman, Isabelle Natalina Fabbricotti, Ruben de Kuyper, Robbert Huijsman

**Affiliations:** Erasmus University Rotterdam, Institute of Health Policy and Management, P.O. Box 1738, 3000 DR Rotterdam, The Netherlands

**Keywords:** Integrated care, Prevention, Frail older people, Effectiveness, Outcome

## Abstract

**Background:**

This study explored the effectiveness of a pro-active, integrated care model for community-dwelling frail older people compared to care as usual by evaluating the effects on a comprehensive set of outcomes: health outcomes (experienced health, mental health and social functioning); functional abilities; and quality of life (general, health-related and well-being).

**Methods:**

The design of this study was quasi-experimental. In this study, 184 frail older patients of three GP practices that implemented the Walcheren Integrated Care Model were compared with 193 frail older patients of five GP practices that provided care as usual. In the Walcheren Integrated Care Model, community-dwelling elderly were pro-actively screened for frailty from the GP practice using the Groningen Frailty Indicator, and care needs were assessed with the EASYcare instrument. The GP practice functioned as single entry point from which case management was provided, and the GP was the coordinator of care. The entire process was supported by multidisciplinary meetings, multidisciplinary protocols and web-based patient files. The outcomes of this study were obtained at baseline, after 3 months and after 12 months and analyzed with linear mixed models of repeated measures.

**Results:**

The Walcheren Integrated Care Model had a positive effect on love and friendship and a moderately positive effect on general quality of life. The ability to receive love and friendship and general quality of life decreased in the control group but was preserved in the experimental group. No significant differences were found on health outcomes such as experienced health, mental health, social functioning and functional abilities.

**Conclusions:**

The results indicated that pro-active, integrated care can be beneficial for frail older people in terms of quality of life and love and friendship but not in terms of health outcomes and functional abilities. Recommendations for future research are to gain greater insight into what specific outcomes can be achieved with proactive and integrated care, considering the specific content of this care, and to allow for the heterogeneity of frail older people in evaluation research.

**Trial registration:**

Current Controlled Trials ISRCTN05748494. Registration date: 14/03/2013.

## Background

The care for community-dwelling frail older people poses a real challenge for health care systems. Due to population ageing, the number of frail older people is increasing rapidly [1]. Furthermore, national health policies are aimed at preventing admission to nursing homes because institutionalization is costly. Frail older people themselves prefer to grow old in the community [2] and want to live independently at home as long as possible; also referred to as ‘ageing in place’ [3]. This could become problematic because frail older people suffer from problems in the physical, psychological and social domains of daily functioning [4]. The quality of care for these frail older people living in the community needs improvement [5]. Currently, care is reactive and the needs of frail older people are not addressed in a timely manner, leading to crisis situations [6]. Care is also fragmented and lacks continuity and coordination [7]. As a way to mitigate these challenges, care for frail older patients in the community should become more pro-active and integrated [8].

Pro-active care for frail older people starts with the identification of this group within the community. Research has shown that frailty is related to negative health outcomes, disability [9], and poor quality of life [10]. To postpone or prevent these outcomes, frailty should be identified quickly and correctly [11, 12]. After the pro-active identification, care should be integrated and delivered coherently according to the needs of the frail individuals related to the areas of prevention, care, cure, housing and welfare [13], meaning that professionals from different disciplines and sectors should collaborate [14, 15]. In the present study, we evaluated the Walcheren Integrated Care Model (WICM), a specific pro-active *and* integrated care intervention aimed at community-dwelling frail older people and implemented in primary care with the GP practice as single entry point and the GP as coordinator of care. This study contributes to the growing body of evidence due to the specific features of the intervention and its extensive evaluation.

WICM is primarily characterized by the combination of a pro-active *and* integrated approach to care for frail community-dwelling patients. Many care interventions for community-dwelling frail older people have a strong focus on integration, but the importance of pro-activeness is not widely acknowledged. In the WICM, frailty is detected from the GP practice by screening the GP’s entire patient population aged 75 years and older. Research has shown that such a pro-active approach, in combination with integrated care elements, is more effective than a pro-active approach alone [16]. Moreover, all integrated care elements that have been recognized to be effective in prior research are included in the WICM instead of considering only a selection of these elements. These elements include the following: geriatric assessments, case management, multidisciplinary teams, a single entry point [17], multidisciplinary protocols and discussions, web-based patient files, and a network structure [15, 18, 19]. This network structure, in which the WICM is embedded, consists of GP practices, home care organizations, nursing homes and patient organizations. The representatives of these involved organizations form the WICM’s Steering Committee, which is an example of organizational integration at the meso-level. This organizational integration is also a specific feature of the WICM because most integrated care interventions are characterized by case management and the relationship between the GP and case manager [20], and integration is restricted to the micro level. The assumption for our approach is that adopting more strategies at different levels is essential to achieve effectiveness [21].

The effectiveness of the pro-active and integrated WICM is evaluated comprehensively by considering an extensive combination of patient outcome measures. Previous evaluation studies have primarily focused on three categories of outcomes corresponding to the three problem areas of frailty: health outcomes, functional abilities and quality of life [16, 22–36]. However, these studies have shown inconsistent results and there is an urgent need for more in-depth evaluation research, in particular for research reporting these three domains simultaneously [25]. Even though no intervention established effects in terms of health outcomes, functional abilities *and* quality of life yet [16, 24], we intended to explore whether the pro-active, comprehensive and highly integrated WICM can achieve effectiveness in all three categories. Hence, this study aimed to answer the following research question: *what is the effect of the WICM on health outcomes (experienced health, mental health, social functioning), functional abilities and quality of life (general, health-related, and well-being) of community-dwelling frail older people?*

## Methods

### Design

The design of this study was quasi-experimental and included before and after measurements with a control group (see also [37]). The measurements were obtained at baseline, after 3 months and after 12 months. The experimental group consisted of older patients of eight GPs from three GP practices located in eastern Walcheren who provided care according to the WICM. The control group consisted of the patients of six GPs from five GP practices who provided care as usual in the northern, southern and western parts of Walcheren.

The study design was reviewed by the medical ethics committee of the Erasmus Medical Center, Rotterdam, the Netherlands, under protocol number MEC-2013-058. This committee waived further examination because the rules established in the Medical Research Involving Human Subjects Act did not apply.

### Participants

**Fig. 1:**
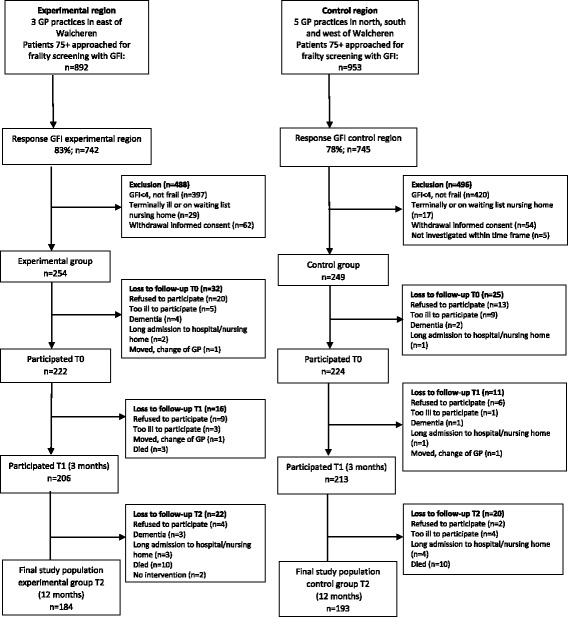
Flow chart of participants

All GP patients aged 75 and older of the 3 GP practices in the experimental (*n* = 892) and 6 GP practices in the control group (*n* = 953) were sent a GFI questionnaire and an informed consent (see Fig. 1). The GFI is a 15-item questionnaire screening for frailty that measures decreases in physical, cognitive, social and psychological functioning. GFI scores range from 0 to 15; patients with a score of 4 or higher were considered frail [38, 39]. In the experimental region 83% of the patients returned the GFI questionnaire; in the control region 78%. Patients were included in the study when they did not fulfil the exclusion criteria of not being frail (GFI score lower than 4); living in a nursing home; being on waiting list for a nursing home; and being terminally ill with a life expectancy under 6 months. At baseline, 254 frail older patients were included in the experimental group, and 249 frail older patients were included in the control group. After 12 months, the final study population included 184 frail older people in the experimental group and 193 frail older people in the control group. Loss to follow-up was mostly caused by frail older people refusing to participate (*n* = 54) or passing away (*n* = 23).

### Intervention

After screening the patient population of each GP with the GFI, frail older patients in the experimental group were visited by a nurse practitioner who assessed their functional, cognitive, mental and psychological functioning using EASYcare. EASYcare is an evidence-based, comprehensive instrument used to assess care needs [31] and has a separate model to translate care needs into specific treatments goals. The GP and nurse practitioner decided on treatment goals in consultation with the older people and their informal caregivers, which were translated into a preliminary multidisciplinary treatment plan. This plan was determined in a multi-disciplinary meeting attended by at least the GP, the nurse practitioner, and a secondary-line geriatric nurse practitioner. Depending on frail elderly's problems discussed, the meeting was also attended by other health professionals such as geriatric physiotherapists, geriatricians, pharmacists, district nurse, nursing home doctors and mental health workers. The concrete actions, activities and responsibilities of these health professionals were discussed during this meeting.

Case management was provided from the GP-practice by the nurse practitioner or by a secondary-line geriatric nursing practitioner, depending on the complexity of the older people person’s problems. The case manager coordinated care within the multidisciplinary team which implies monitoring the frail older person’s condition, arranging the admittance to the required services, being the contact person for the involved professionals to coordinate their care and periodically evaluating the multidisciplinary treatment plan. The evaluation occurred in multidisciplinary meetings. The entire process was supported by web-based patient files and multidisciplinary protocols describing the responsibilities and activities of the involved professionals, in particular the nurse practitioner and secondary-line geriatric nursing practitioner who provided case management. Protocols were also designed for common themes such as incontinence, polypharmacy, and falling. In the WICM, the GP had the final responsibility and functioned as a coordinator of care and partner in prevention. The GP practice was a single entry point for the older frail patients, their informal caregivers and health professionals where they could gain access to information and services of all involved professionals and organizations.

The model required task reassignment and delegation between nurses and doctors and among GPs, nursing home doctors and geriatricians. Consultations among primary, secondary, and tertiary care providers occurred. Regarding integration at the organizational level, a Steering Committee serves as an umbrella organization under which the WICM is developed and disseminated. The Steering Committee consists of representatives from all involved organizations, such as GP practices, home care organizations and nursing homes, and provides the necessary provider network. Patient representatives support the project, and the health insurer CZ provides financial support for the project.

### Care as usual

Compared with the WICM, care as usual in the Netherlands is reactive and fragmented (Table 1). Every Dutch citizen is registered at a particular GP practice (or family doctor) near their home. Dutch patients first consult their GP for all health problems. GPs play the role of gate keeper [40], patients must have a referral from their GP to obtain care from the primary, secondary and tertiary echelons [13]. However, patients solely receive care for specific (health) problems on their own initiative.

**Table 1:** Differences between WICM and care as usual

	WICM	Care as usual
Role GP	Single entry point, coordinator of care	Gatekeeper
Pro-activeness versus reactiveness	Entire patient population of 75+ is screened for frailty	Patients receive care on their own initiative
	Comprehensive assessment of care needs with EASYcare	Patients receive care for specific health problems
Treatment plan	Multidisciplinary treatment plan	No or monodisciplinary treatment plan
Care coordination	Case management: monitoring, admittance to services, contact person for professionals, evaluating treatment plan	No case management
Communication	Multidisciplinary meetings and web-based files	Bilateral communication by phone calls and letters
Protocols	Multidisciplinary protocols	Monodisciplinary protocols
Network	Network structure	No participation in provider network

Care as usual is fragmented and has a mono-disciplinary focus. Even though the GP is a generalist and has the role of gatekeeper, communication between professionals from the different disciplines and sectors is bi-lateral through referral letters and sporadic telephone calls.

The GPs in the control group were unable to implement elements of the integrated model during the study period because they did not receive financial support from the health insurer to implement the integrated care activities of the WICM. Furthermore, the GPs in the control group could not treat frail older patients differently, as these GPs were not given information on who participated in the study. Therefore, the probability of bias was minimized [41].

### Data collection and measures

Data was collected with questionnaires at three points in time: at baseline, after 3 months and after 12 months. All older people were visited at home by trained interviewers recruited from the region of Walcheren to ensure a cultural fit with the frail older people. Interviewers had a background in healthcare to ensure a high-quality interview.

Health, functional abilities and quality of life were studied, primarily with validated instruments. All health outcomes (experienced health, mental health and social functioning) were assessed by means of questions from the RAND-36 questionnaire [42]. Experienced health was assessed with one item from the RAND-36 that allows frail older people to evaluate their own health. Mental health was measured using a five-item RAND-36 scale with items that question how often the respondent feels certain emotions, such as happiness or nervousness; the Cronbach’s alpha for this scale was 0.74. Social functioning was measured with one item that asked how often social activities were hampered by physical health or emotional problems.

Functional abilities were measured with the Katz-15 instrument that assesses the ability to perform 15 activities of daily living, such as getting dressed, shopping and taking medication [43]; the Cronbach’s alpha of this instrument was 0.86.

To assess quality of life, various instruments were used. First, a general measure of quality of life was used, which was based on the RAND-36 [42]. The second measure was the EQ-5D, which focuses on health-related quality of life and includes five dimensions: mobility, self-care, daily activities, pain/discomfort and mood [44, 45]. The third measure was the ICECAP, which was specifically developed to assess the quality of life related to older people well-being. The ICECAP measures five dimensions of quality of life: attachment, security, role, enjoyment and control [46]. This instrument is based on Sen’s capability approach, which focuses on whether older people are able to function within these domains [47]. All outcome variables are continuous and measured at the interval level.

The covariates included are age, gender, marital status (0: married and living together; 1: single and widowed), living arrangement (0: independently; 1: assisted living facility) and educational level (0: low; 1: high). Age is a continuous variable measured at the ratio level and all other covariates are categorical variables measured at the nominal level.

### Statistical analysis

The study population was described, and baseline differences between the experimental and control groups were tested using chi square tests for categorical variables and independent t-tests for continuous variables. Each outcome variable after 3 and 12 months of follow-up was analyzed with linear mixed models of repeated measures. In all models, time and intervention (experimental and control group) were included and we adjusted for the baseline score of the specific outcome variable and for the covariates sex, age, marital status, educational level, and living arrangement. The significance level was set at *p* < 0.05 and p-values of <0.10 were also reported [48]. All analyses were performed with SPSS 22.

## Results

The study population consisted of frail older patients with an average age of 82 years and an average score of 6 on the GFI (see Table 2). Women were overrepresented in both groups: 70% of the experimental group and 60% of the control group were female. Sixty-three percent of the frail older people in the experimental group and 47% in the control group had a lower level of education. The majority of the frail older people did not have a partner and lived independently. Frail older people reported on average four morbidities; most common were joint damage, hearing problems, vision disorders and heart failure.

**Table 2:** Baseline characteristics of the study population

	Experimental group (*n* = 184) Mean (SD) or %	Control group (*n* = 193) Mean (SD) or %	p-value
Background variables			
GFI (0–15)	6.0 (2.0)	5.8 (1.8)	0.19
Age	81.8 (4.7)	82.3 (5.3)	0.38
Sex – women	69.6%	59.6%	0.04
Educational level Low High	63.0% 37.0%	46.6% 53.4%	0.00
Marital status Married and living together Single and widowed	37.0% 63.0%	41.7% 58.3%	0.35
Living situation Independently Assisted living facility	71.7% 28.3%	82.4% 17.6%	0.01
Multimorbidity	3.8 (1.9)	3.9 (1.9)	0.66
Outcomes			
Health outcomes			
Experienced health (0–100)	33.8 (17.1)	35.1 (20.5)	0.51
Mental health (0–100)	71.3 (17.6)	72.0 (16.5)	0.69
Social functioning (0–100)	69.1 (33.7)	65.7 (39.0)	0.36
Functional abilities			
Functional abilities (0–15)	3.9 (3.1)	3.7 (3.2)	0.48
Quality of life			
General quality of life (0–100)	42.3 (18.0)	47.0 (19.4)	0.01
Health-related quality of life (0–1)	0.6 (0.2)	0.7 (0.3)	0.60
Well-being – love & friendship (1–4)	3.1 (0.8)	3.0 (0.8)	0.20
Well-being – security (1–4)	3.2 (0.9)	3.3 (0.8)	0.32
Well-being – role (1–4)	2.7 (0.8)	2.8 (0.8)	0.12
Well-being – enjoyment (1–4)	3.0 (0.8)	2.8 (0.8)	0.08
Well-being – control (1–4)	2.6 (0.9)	2.8 (0.9)	0.08

Compared with the control group, the experimental group consisted of significantly more women, more less-educated individuals and more individuals residing in assisted living facilities.

The results at baseline showed that frail older people find their mental health and social functioning to be less problematic than their health. The average score on functional abilities was approximately 4, meaning that frail older people need help with 4 (instrumental) activities of daily life. The score for health-related quality of life was approximately 0.6, and the scores on the domains of well-being ranged from 2.6 to 3.2. At baseline, health outcomes, functional abilities and quality of life were equal in both groups, except for general quality of life. General quality of life was significantly lower at baseline in the experimental group than in the control group (42.3 vs. 47.0, *p* < 0.05).

Table 3 shows that the WICM had limited effects on health outcomes, functional abilities and quality of life. The WICM had a moderate significant effect on quality of life after 12 months (CI: (−0.15 to 5.63; *p* < 0.10). Whereas the general quality of life of the frail older people in the control group decreased over 12 months, the quality of life of the frail older people in the experimental group was preserved. With regards to health-related quality of life and well-being, no effects were found. However, WICM impacted one dimension of well-being: the ability to receive love and friendship (CI: (0.14 to 0.36; *p* < 0.001). In the control group, the ability to receive love and friendship decreased, but this ability did not change in the experimental group. No significant differences were found between the groups in terms of experienced health, mental health and social functioning. Moreover, functional abilities of frail older people were not affected by the WICM.

**Table 3:** Linear mixed models – adjusted overall effects^a^

	Mean (SE) experimental	Mean (SE) control	Mean diff (95% CI)	p-value
Outcomes				
Health outcomes				
Experienced health (0–100)	34.31 (1.01)	34.99 (1.04)	−0.68 (−3.18 to 1.82)	0.59
Mental health (0–100)	68.86 (0.94)	69.44 (0.91)	−0.42 (−2.69 to 1.85)	0.72
Social functioning (0–100)	65.06 (2.29)	66.42 (2.36)	−1.36 (−7.04 to 4.33)	0.64
Functional abilities				
Functional abilities (0–15)	4.41 (0.14)	4.19 (0.14)	0.22 (−0.13 to 0.56)	0.21
Quality of life				
General quality of life (0–100)	42.66 (1.15)	39.92 (1.19)	2.74 (−0.15 to 5.63)	0.06
Health-related quality of life (0–1)	0.66 (0.01)	0.65 (0.02)	0.01 (−0.03 to 0.04)	0.73
Well-being – love & friendship (1–4)	3.00 (0.04)	2.75 (0.05)	0.25 (0.14 to 0.36)	0.00
Well-being – security (1–4)	3.32 (0.05)	3.28 (0.06)	0.05 (−0.08 to 0.18)	0.45
Well-being – role (1–4)	2.57 (0.05)	2.54 (0.05)	0.03 (−0.10 to 0.15)	0.66
Well-being – enjoyment (1–4)	2.73 (0.05)	2.66 (0.06)	0.07 (−0.06 to 0.19)	0.30
Well-being – control (1–4)	2.55 (0.05)	2.61 (0.05)	−0.07 (−0.19 to 0.06)	0.27

^a^Adjusted for the baseline score of the specific outcome variable, sex, age, marital status, educational level, and living arrangement

All baseline scores were strongly significant and were the main determinant for all outcomes after 12 months. Of the covariates, age was the most important and had a negative effect on social functioning, functional abilities, and health-related quality of life. Marital status had a negative effect on two outcomes, as frail older people with a partner showed lower scores for social functioning and functional abilities. In addition, two significant trends over time could be observed: functional abilities and health-related quality of life both decreased over time.

## Discussion

In this study, we explored the effectiveness of the WICM in terms of health outcomes, functional abilities and quality of life. The WICM is an intervention that combines a pro-active and integrated care approach organized from the GP practice; the model contains diverse effective integrated care elements, and integration is achieved at the organizational level. Our study shows that the WICM has a positive effect on the ability to receive love and friendship, and the WICM moderately preserves the general quality of life of frail older people. The WICM is not effective in terms of health outcomes and functional abilities.

The effect of the WICM on quality of life could possibly be explained by the pro-active approach of the WICM and its target group. Previous research has shown that a pro-active attitude has positive results on quality of life [10] and that timely identification of frailty prevents further deterioration [11, 12]. Moreover, in the WICM, older people are pro-actively screened for frailty from the GP practice with the GFI questionnaire, which strongly determined the target group for the intervention. The GFI questionnaire was sent to *all* GP patients aged 75 years or older and focuses on physical, cognitive, social and psychological functioning [38, 39]. Compared to other interventions, in which quality of life was considered an outcome variable, our study had a broader approach to frailty and therefore a different target group. In other interventions, older people were included in the interventions if they reported having problems [30, 31], visited the emergency department [27], were referred by family practitioners [34] or were screened by routine care data [16]. Accordingly, the differences in target groups between the interventions could possibly explain the difference in outcomes.

The WICM also had an effect on love and friendship, which are two important attributes of the quality of life of elderly [47]. Previous evaluation research on the short-term effects of the WICM also showed this effect [49], which indicates the consistency of this relevant finding. This consistent effect may be explained by the improved relationship between frail older people and their informal caregivers. In the WICM, the situation of frail older people is comprehensively assessed and monitored in consultation with the informal caregiver, possibly leading to tranquility and relief. This notion is underscored by the finding that the WICM had a positive effect on the subjective burden of the informal caregivers [50]. The informal caregivers indicated that their caregiver situation improved in terms of, for example, mental health and relationships, which could have affected the feelings of love and friendship experienced by frail older people.

Furthermore, the WICM did not show effects on health outcomes and functional abilities. Integrated care interventions such as the WICM, encompass the reorganization of care processes targeting at multi-dimensional needs of persons with similar problems [51]. However, this does not provide insight in the specific content of these care processes. Reorganization of care for frail older people might not be sufficient to achieve effectiveness in terms of health outcomes and functional abilities. The content of care might also be important; research has shown that integrated care containing specific medical and paramedical interventions has resulted in positive outcomes for frail older people [29, 52]. With respect to medical and paramedical care, the differences between WICM and care as usual were limited, given that the Netherlands has a strong primary care system. An important distinction between WICM and care as usual is the multidisciplinary focus. The care in WICM is not purely medical but also entails prevention, residence and wellbeing. WICM’s primary outcome measure was, therefore, quality of life [37].

### Strengths

The strength of our study was its consideration of many different outcomes, which were measured with innovative instruments such as the ICECAP. The ICECAP instrument has been developed to measure older people well-being, even when personal functioning is not improving [53]. This instrument covers the five most important attributes of older adults’ well-being, including love and friendship [46]. The effectiveness of integrated care has not been examined previously with this specific instrument. However, the ICECAP has been used in economic evaluations, in which it was shown that this instrument is more sensitive at detecting the effectiveness of interventions for frail older people than the EQ-5D-instrument, a more traditional instrument to measure health-related quality of life [54].

### Limitations

The primary limitation of our research is that the design of the study was quasi-experimental. To ensure that frail older people could receive care from their own GP, randomization of the frail older people population was not desirable. Our quasi-experimental design, however, means that the study population in the experimental and control groups could have differed non-randomly at baseline. In our study, the experimental group consisted of more women, more individuals living in assisted living facilities and more individuals with a lower level of education. However, these differences may not have impacted our results for two reasons. First, we accounted for these differences by including the background characteristics as covariates in our analyses. In these analyses, no significant effects were found for sex, living situation and educational level. Second, previous research has not shown consistent effects of these variables on factors such as quality of life [55, 56].

A second limitation is our focus on patient outcomes. Even though a comprehensive set of outcome measures was used in terms of health, functional abilities and quality of life, the effects of WICM on health care utilization remain to be determined. Integrated care has been shown to result in a decline in hospitalization and institutionalization [25]. Therefore, it would be useful to explore whether our integrated and pro-active intervention would affect health care utilization and associated costs. These costs could be compared with the effects of our intervention, such as health-related quality of life, to allow for statements regarding the cost-effectiveness of the WICM.

### Recommendations

Recommendations for practice are that more in-depth insights into the effectiveness of preventive and integrated care approaches for frail older patients are required. Integrated care interventions such as the WICM should be further optimized in practice; it still remains unclear what specific combinations of pro-active *and* integrated care elements are most effective. The comprehensive WICM pursuing integration at the micro- and meso-level with a preventive focus showed moderate positive results in terms of quality of life but this intervention was not able to improve health outcomes and functional abilities. Furthermore, our study revealed that the specific content of care within these integrated care interventions for community-dwelling elderly should be carefully considered in the future development of these interventions including the WICM.

Regarding the outcomes for frail older people, future research is recommended to explore what specific outcomes could be expected for frail older people and how these outcomes could be accurately detected in evaluation research. Frailty is a gradual process of deterioration [4], and it might not be realistic to expect improvement or even preservation in all three domains (i.e., health, functional abilities and quality of life). However, our study shows that a slightly different emphasis, for example, by examining specific domains of well-being, is encouraging. In particular, the ICECAP instrument is recommended for inclusion in future evaluation research.

The final implication of this study for future research is enhancement of our understanding of the participants of integrated care interventions. Although all participating older people in the various studies have been described as frail, inclusion criteria or screening instruments to detect frailty in these studies were different (see also [25]). In addition, thus far, frail older people have been perceived as a single group in classical evaluation studies; no distinction of any kind has been made among frail older people, even though research has shown that they are a heterogeneous group of people with diverse problems in physical, psychological and social domains [4]. This heterogeneity should also be considered in the evaluation of integrated care and may possibly yield insight into its effectiveness.

## Conclusions

The conclusion is that WICM, a pro-active and integrated care intervention with the GP-practice as single entry point, is moderately effective for community-dwelling frail older people. WICM had a positive effect on the ability to receive love and friendship and moderately preserves general quality of life; two relevant findings because they comprise the personal evaluation of the frail older people themselves. However, WICM was not effective in terms of health outcomes and functional abilities.

## Abbreviations

GFI: Groningen Frailty Indicator
GP: general practitioner
WICM: Walcheren Integrated Care Model
